# A spatial numerical model for seagrass–herbivore interactions and the formation of reef halos

**DOI:** 10.1007/s00338-025-02729-3

**Published:** 2025-08-20

**Authors:** Eva Llabrés, Anne A. Innes-Gold, Bartholomew DiFiore, Tomàs Sintes, Elizabeth Madin

**Affiliations:** 1 Institute for Cross-Disciplinary Physics and Complex Systems (IFISC), CSIC-UIB, 07122 Palma de Mallorca, Spain; 2 Hawai’i Institute of Marine Biology, University of Hawai’i at Mānoa, Kāne’ohe, HI 96744 USA; 3 Gulf of Maine Research Institute, Portland, ME 040101 USA

**Keywords:** Herbivory patterns, Seagrass meadows, Species interactions, Spatial ecology, Agent-based modeling, Remote sensing

## Abstract

Reef halos are circular patterns of bare sand surrounding patch reefs, formed by herbivorous fish grazing near their reef refuges. These formations serve as indicators of ecological processes, providing insights into interactions among herbivores, vegetation, and predators. Their size and prevalence are influenced by predator and herbivore densities, fishing pressure, and temperature, making them valuable proxies for assessing the impact of anthropogenic stressors on reef ecosystems. Halos can also be monitored using satellite imagery and artificial intelligence tools, offering a scalable method for evaluating ecosystem health. In this study, we present the first spatially explicit agent-based model to explore reef halo formation. By integrating seagrass growth models with herbivorous fish behavior, we capture the spatial complexity of halo dynamics. Our model reproduces observed field patterns, including halo size variability driven by temperature. In addition, the model uncovers new insights into the mechanisms behind the formation of sand corridors—vegetation-free pathways that link isolated halos—an aspect of halo dynamics that was previously unresolved. We propose that these corridors are shaped by limitations in rhizome growth rather than shifts in herbivore foraging behavior. These findings advance our understanding of the ecological processes driving halo formation and enhance the predictive value of halos as indicators of coral reef ecosystem health. The model offers a deeper insight into how reef systems respond to environmental pressures, providing a powerful tool for monitoring and managing reefs amid climate change and anthropogenic impacts.

**Supplementary Information:**

The online version contains supplementary material available at 10.1007/s00338-025-02729-3.

## Abstracta

En este trabajo presentamos el primer modelo explícitamente espacial que aborda la formación de halos en arrecifes de coral. Al integrar el crecimiento de las praderas marinas con el comportamiento de los peces herbívoros, capturamos la complejidad espacial de la dinámica de los halos y reproducimos patrones observados en campo, incluida la variación del tamaño en función de la temperatura. Además, el modelo arroja luz sobre la formación de corredores de arena—pasillos sin vegetación que conectan halos aislados— un fenómeno del que hasta ahora se desconocían su causa. Sostenemos que estos corredores se originan por limitaciones en el crecimiento rizomatoso, más que por cambios en la alimnetación de los herbívoros. En conjunto, los resultados avanzan en la comprensión de los procesos ecológicos que moldean los halos y refuerzan su utilidad como indicadores del estado de los ecosistemas en arrecifes de coral. El modelo ofrece, además, una base predictiva para evaluar la respuesta de los halos a las presiones ambientales y orientar su seguimiento y gestión frente al cambio climático y las actividades humanas.

## Introduction

Reef halos are visually striking patterns observed in underwater meadows, characterized by rings of bare sand surrounding patch reefs (Randall 1965; Ogden et al. 1973). These formations have been widely observed across marine ecosystems, with a long history of documentation (Bilodeau et al. 2021). The most widely accepted hypothesis for the formation of these halos is based on herbivory: fish graze in the vicinity of patch reefs, where they find refuge from predators, creating a gradient in grazing intensity that produces the characteristic halos (Randall 1965; Gil et al. 2017; Madin et al. 2011; Downie et al. 2013). As a result of these dynamics, reef halos function as macroscopic indicators of ecological processes, offering insights into how interactions among herbivores, vegetation, and predators shape habitats on broader spatial scales. The shape, size, and prevalence of halos can vary according to key environmental factors, such as fishing pressure or herbivore abundance per unit area (Madin et al. 2019a, b; DiFiore et al. 2019), making them a useful proxy for assessing the impact of anthropogenic stressors on reef ecosystems. Furthermore, because halos can be efficiently monitored using satellite imagery and artificial intelligence (AI) tools, they offer a scalable, cost-effective method for evaluating ecosystem health, potentially serving as an early warning for environmental threats like overfishing and climate change (Madin et al. 2011, 2022; Franceschini et al. 2023).

Halo patterns are commonly found within seagrass and algal meadows around the world (Randall 1965; Hay 1984; McAfee and Morgan 1996; Madin et al. 2022). Seagrasses, and some algae, propagate primarily through clonal growth, driven by horizontal stems called rhizomes that extend beneath the sediment. These rhizomes periodically branch and produce new shoots, enabling seagrasses to form dense, interconnected meadows that expand across extensive areas. This vegetative reproduction not only facilitates habitat creation for marine species, but also stabilizes sediments and serves as a critical food source for herbivores (Valentine and Heck 1999). The concept of clonal growth has been central to the development of mathematical models that have significantly advanced our understanding of seagrass dynamics (Sintes et al. 2005; Ruiz-Reynés et al. 2017). For instance, these models have been instrumental in exploring self-organized pattern formation (Ruiz-Reynés et al. 2017; Ruiz-Reynés and Gomila 2019), predicting future seagrass populations under global warming scenarios (Llabrés et al. 2023), and evaluating their potential as carbon sinks (Duarte et al. 2013). By integrating empirical data with theoretical frameworks, these models provide invaluable insights into the ecological roles of seagrasses and their responses to environmental changes.

Existing models of seagrass–herbivore interactions have primarily focused on non-spatial consumer-resource dynamics, leveraging geometric considerations within the partial differential equations to still gain insights into halo distribution (Ong et al. 2025). However, reef halos are inherently spatial phenomena, arising from localized herbivory that disrupts seagrass growth near reef refuges. This spatial complexity produces unique patterns that non-spatial models cannot fully capture. One such pattern is grazing corridors (Madin et al. 2022)—linear vegetation-free paths that connect halos—breaking the typical radial symmetry central to previous models. The formation of these grazing corridors exemplifies how spatially explicit approaches are essential for capturing the full complexity of halo dynamics. Additionally, while herbivory is the primary driver of halo formation, abiotic factors such as temperature fluctuations and nutrient availability also play a key role in halo size with their specific effects differing across ecosystems (Innes-Gold et al. 2025; Lester et al. 2025). A unified spatial framework that accounts for both biological and environmental drivers would not only improve our understanding of halo dynamics but also provide a powerful tool for linking changes in halo size to specific ecological stressors, ultimately supporting efforts to assess and protect reef ecosystem health (Bilodeau et al. 2021).

In this study, we develop a unique numerical framework that captures the key ecological interactions responsible for the formation of reef halos. We introduce a novel, spatially explicit model that couples existing seagrass growth models with herbivorous fish behavior, providing a comprehensive view of landscape-scale dynamics. Our model is designed to reproduce observed field patterns, such as varying halos sizes, while providing a robust theoretical framework to explore the ecological processes and functional groups that shape these formations. To showcase its utility, we present two key applications: first, we demonstrate the model’s ability to predict variability in halo size as a function of temperature and herbivore abundance per unit area, which may prove valuable for assessing the impacts of climate change on reef ecosystems. Second, the model provides new insights into the mechanisms underlying the formation of sand corridors that link otherwise isolated halos, addressing previously unresolved questions about halo formation dynamics. These examples highlight the model’s dual utility in both evaluating ecosystem health and advancing our understanding of the fundamental drivers of halo formation.

## Methods

### Numerical model

Here, we present a novel agent-based model designed to reproduce landscape patterns of reef halos by incorporating interactions between seagrasses, herbivores, predators, and coral reefs. The model operates in two-dimensional space and is structured on a lattice with a resolution of $r_c$. Coral reefs are treated as boundary conditions—areas where herbivores reside and seagrasses cannot grow. Reef growth is not explicitly considered in the model, as it occurs on timescales much longer than those of seagrasses and fishes. Seagrasses are modeled using a set of empirically derived rules based on their clonal growth, as proposed by Sintes et al. (2005). These rules are also based on measurable parameters and summarized as follows. Apices, the growing tips of the rhizome, drive horizontal propagation at a constant elongation rate of $v \, [\text {cm} \cdot \text {yr}^{-1}]$. Along the rhizome, shoots emerge at regular intervals, spaced on average by $\delta \, [\text {cm}]$, while branching occurs with probability $\nu _0 \, [\text {yr}^{-1}]$, with an angle $\theta \,[^\circ ]$. Shoot mortality rates, $\omega \, [\text {yr}^{-1}]$, vary with local environmental conditions. The model also incorporates local seagrass interactions by adding a density-dependent branching rate (Llabrés et al. 2022), i.e., $\nu (S)=\nu _0 + \hat{S} \left( 1-\hat{S} \right) \cdot \nu _0/2$, where $\hat{S} = S /S_{\max}$ is the normalized local seagrass density. These growth rules have been extensively validated and shown to effectively simulate seagrass meadows. For further details, we refer to the original models and their results (Sintes et al. 2005, 2006; Llabrés et al. 2022, 2023).

Building upon this clonal model, we propose a novel framework that couples seagrasses growth to herbivore behavior, capturing the interactions between both populations. While seagrass shoots remain stationary, herbivores move dynamically across the spatial domain. To account for this movement, we model fish positions probabilistically using Gaussian distribution, expressed as follows

1 $$\begin{aligned} p_i (\textbf{x}) = {1\over 2\pi \sigma ^2} e^{-{(\textbf{x}-\textbf{x}_i)^2\over 2\sigma ^2}}, \end{aligned}$$

where $p_i (\textbf{x})$ represents the likelihood of a fish labeled by *i* being at a given 2D position $\textbf{x}$. The origin of the Gaussian distribution at $\textbf{x}_i$ corresponds to the fish’s fixed refuge at the reef perimeter. This formulation is motivated by empirical observations that fish remain close to reefs and utilize more or less fixed areas for shelter (Shabi et al. 2024; Ceccarelli et al. 2001; Kane et al. 2008). The parameter $\sigma$ defines the dispersion of the Gaussian distribution, and reflects the herbivores’ willingness to venture away from the reef. In nature, distance from shelter scales with the risk of consumption by a predator (Brown and Kotler 2004), thus venturing farther from shelter increases an individual’s risk of being consumed if all else is equal. The dispersion parameter $\sigma$ is therefore a proxy for predator abundance in that a larger $\sigma$ suggests reduced perceived predation risk, and thus a willingness of herbivores to venture farther from shelter, whereas a smaller $\sigma$ indicates a stronger ’fear effect’ caused by higher predator density and thus greater predation risk. Our assumptions in Eq. (1) parallel those made in Madin et al. (2010), where fishes are modeled as performing 1d random walks from the reef, though in our case, we extend the spatial consideration to two dimensions.

Following this characterization of herbivore movement, our overall aim is to describe the dynamics of the herbivore population and their effects on seagrass meadows. We identify two primary mechanisms that drive these interactions: (a) herbivores control seagrass through grazing, concentrating their foraging near reefs and forming reef halos, and (b) seagrass abundance influences herbivore reproduction, with resource availability supporting population growth. To incorporate these dynamics, we define the following conditions:

**(a)**: **Seagrass mortality due to grazing:** The rate of shoot mortality at $\textbf{x}$ due to grazing is denoted by
  2 $$\begin{aligned} \mathcal{F}(\textbf{x}) = {\alpha \over 2\pi \sigma ^2}\sum _{i\in N_H} e^{-{(\textbf{x}-\textbf{x}_i)^2\over 2\sigma ^2}}, \end{aligned}$$
  where the sum runs over the total number of herbivorous fishes $N_H$, and each $\textbf{x}_i$ represents the coordinates of their respective refuges at the reef perimeter. The expression is derived from the idea that $\sum _i p_i({\textbf{x}})$ reflects the likelihood of encountering a fish at the coordinate $\textbf{x}$, as indicated by Eq. (1). Consequently, when this probability is multiplied by the individual grazing rate $\alpha$ $[\text { shoots grazed}\cdot \text {day}^{-1}\cdot \text {fish} ^{-1}]$, it yields the mortality rate resulting from grazing. For simplicity, we assume that the grazing rate $\alpha$ is uniform across all herbivores.
**(b)**: **Fish reproduction due to resource abundance:** The functional $\mathcal{G}(\textbf{x}_i)$ represents seagrass availability to an herbivore with refuge at $\textbf{x}_i$:
  3 $$\begin{aligned} \mathcal{G}(\textbf{x}_i) = {1\over 2\pi \sigma ^2 }\int d\textbf{x}' e^{-(\textbf{x}'-\textbf{x}_i)^2\over 2\sigma ^2} {S(\textbf{x} ')}, \end{aligned}$$
  where *S*(**x**') is the normalized seagrass density ($$S = S/S_{\max}$$). The integral sums the seagrass distribution $$S(\textbf{x}')$$ across the herbivore’s foraging range, weighting each location  by the probability  $$p_i(\textbf{x}')$$ of the herbivore being there (Eq. 1). In our simulations, the probability of reproduction for an herbivore at $\textbf{x}_i$ is 
  4 $$\begin{aligned} { \mathcal P}(\textbf{x}_i) = \gamma \, \mathcal{G}(\textbf{x}_i)\left( 1- {H(\textbf{x}_i)\over H_{\max}}\right) , \end{aligned}$$
  where $\mathcal{G}$ represents the seagrass availability as in Eq. (3), and the term $(1- H/H_{\max})$ serves as a carrying capacity, restricting the herbivore population to a maximum of $H_{\max}$. The parameter $\gamma \,[\text {yr} ^{-1}]$ determines the strength of the herbivore–seagrass interaction, and reflects the probability of a fish reproducing if it had all the available resources. Additionally, we consider a natural mortality rate $\mu \,[\text {yr} ^{-1}]$ of the herbivores, acknowledging their limited longevity.

These interactions are well-established in population dynamics, especially in consumer-resource models like those of the Lotka–Volterra family, which are typically formulated using time-dependent partial differential equations (Murdoch et al. 2013). The novelty of our model lies in the incorporation of these interactions into an agent-based framework that explicitly accounts for spatial dependence. This spatial framework allows us to better capture the heterogeneity of seagrass meadows and the distribution of herbivores, leading to the emergence of patterns in the seascape, such as reef halos.

### Field data and model parameters

**Fig. 1:**
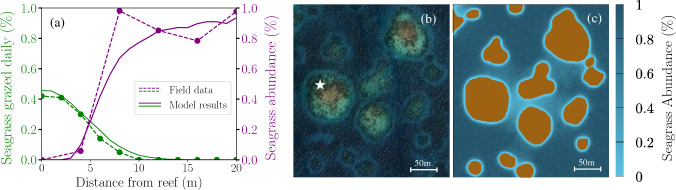
Comparison of model simulations and field observations of reef halos in patch reefs in Carrie Bow Caye, Belize. Field data was collected by DiFiore et al. (2019) and used to parametrize our model. **a** Observed (dashed lines) and modeled (solid lines) daily seagrass grazing (green) and seagrass abundance (purple) averaged over three transects perpendicular the patch reef marked in panel (**b**) with a white star. In the model, seagrass abundance is represented as a density normalized by a maximum value of 4000 m$^{-2}$, while in the field experiments, is measured as a percentage of cover. **b** Satellite image with coordinates $16^\circ \; 45^{\prime }\;02.4^{\prime \prime }\;N, \, 88^\circ \;05^{\prime }\;53.0^{\prime \prime }\;W$, retrieved from *Google Earth* (imagery date: Jan. 2024). **c** Simulation of grazing patterns around patch reefs in Belize using our model. The color bar represents percentage of seagrass cover with colors ranging from light blue (low density) to dark blue (high density). Patch reefs are depicted in brown. Parameters for seagrass growth are based on *T. testudinum* and detailed in Table S1, while herbivore parameters are fixed as in Table S2, with $\gamma =0$ for constant herbivore density

A critical step in developing numerical models for ecological systems is the selection of appropriate parameters. For seagrasses, these parameters are relatively straightforward to measure, since seagrasses are rooted and stationary. Comprehensive datasets for most existing species have already been compiled in studies such as Marbà and Duarte (1998). In contrast, quantifying fish behavior presents a greater challenge due to their mobility. However, a standard method for measuring grazing activity in herbivory studies involves placing baits at regular intervals along a transect perpendicular to the reef and retrieving them after a set period to quantify grazing rates (Hay 1984). This provides the rate of seagrasses grazed as a function of distance from the reef. To compare this field data with the parameters in our model, we must determine how the two-dimensional spatial grazing modeled in step (a) influences a one-dimensional transect. Fortunately, we can reduce the sum in Eq. (2) to:

5 $$\begin{aligned} \mathcal{F}(d) \sim \quad {\alpha \cdot \lambda _{H}\over \sqrt{2\pi }\sigma }e^{-{{d}^2\over 2\sigma ^2}} , \end{aligned}$$

where *d* is the distance from the reef, and $\lambda _{H} \,[\text {m}^{-1}]$ represents the linear density of fish along the reef perimeter. It is related to the herbivore density *H* by $\lambda _{H} = H \cdot {area / perimeter}$, where *area* and *perimeter* refer to the corresponding measurements of the patch reef that serves as the herbivores’ refuge. The derivation of Eq. (5) is not trivial and valid only in the regimes $R>> \sigma$, and $r_c<< \sqrt{2}\sigma$ (for details, see Supplementary Materials S1). This expression is particularly useful as it provides a simplified, yet effective, characterization of fish grazing behavior. Using field data on fish grazing averaged over multiple transects, along with an estimate of herbivorous fish density, we can fit the observed grazing pattern to Eq. (5) to extract two key model parameters: the individual grazing rate $\alpha$ and the dispersion parameter $\sigma$. This approach is applied in “Reproducing field data and satellite imagery” section, where we parametrize the model using grazing field data from Belize, collected by DiFiore et al. (2019) and represented by the green dashed line in Fig. 1a. By fitting this data to the Gaussian function in Eq. (5), we obtain a standard deviation of $\sigma = 3.98 \, \text {m}$ and a grazing rate of $\alpha = 7.6 \, \text {shoots grazed} \cdot \text {day}^{-1} \cdot \text {fish}^{-1}$, when assuming a herbivore density of $\lambda _H = 0.60 \, \text {m}^{-1}$.

In this study, we also investigate the response of reef halos to seasonal variability. Previous work has documented the decline of certain seagrass species, such as *Posidonia oceanica* and *Halophila stipulacea*, in response to increasing temperatures (Savva et al. 2018; Marbà and Duarte 2010; Wesselmann et al. 2021). Building on these findings, we focus on *Halophila hawaiiana*, whose shoot mortality we model as:

6 $$\begin{aligned} \omega (T) = a (T- T_0) + \omega _0 \end{aligned}$$

where *T* is the surface sea water temperature, and $a = 0.45\,\text {yr}^{-1}\cdot ^\circ \hbox {C}^{-1}$, $\omega _0 = 8\,\text {yr}^{-1}$, and $T_0 = 24^\circ \hbox {C}$. This equation is based on data collected by Innes-Gold et al. (2025) in Hawai’i, showing a linear decline in seagrass density with increasing temperature (see Supplementary Materials S2 for more details). The relationship expressed in Eq. (6) is valid within the temperature interval of $24^\circ \hbox {C}$ to $28^\circ \hbox {C}$, consistent with the range of the measurements. In our simulations, herbivore biomass is held constant ($\gamma = 0$) due to the absence of a significant correlation with temperature in field observations (Fig. S2b).While direct data on grazing intensity were unavailable, herbivore foraging behavior was set to maintain halo sizes consistent with field observations from Innes-Gold et al. (2025). To achieve this, we set the parameters to $\sigma = 0.398\, \text{m}$ and $\alpha = 0.138 \text { shoots grazed}\cdot \text {day}^{-1}\cdot \text {fish}^{-1}$.

In this work, we study three geographical regions where reef halos form within seagrass meadows: Carrie Bow Caye in Belize, Kāne’ohe Bay in Hawai’i, and Ras Mohammad National Park in Egypt. The dominant seagrass species at each of these locations are *Thalassia testudinum*, *Halophila hawaiiana*, and *Halodule uninervis*, respectively (DiFiore et al. 2019; Innes-Gold et al. 2025; Yasser 2009). The seagrass model parameters for each site were fixed based on data from Marbà and Duarte (1998) and are detailed in Table S1. Additionally, we provide a summary of key herbivore parameters and their values detailed in Table S2. Moreover, in the case of Belize and Egypt, the reefs we model correspond to real reef structures at these locations. Their shapes were mapped using ArcGIS to generate shapefiles, and processed used the Python library GeoPandas to convert them into an array that identifies which lattice cells are occupied by the reef. In the case of Hawai’i, we incorporate 1x1m structures in our simulations to mimic the artificial reefs used in the field experiments carried by (Innes-Gold et al. 2025).

## Results

### Reproducing field data and satellite imagery

Simulations generated by our model closely replicate the observed reef halo patterns around patch reefs, as shown in Fig. 1. The green dashed line in Fig. 1a represents grazing rates measured from Carrie Bow Caye, Belize, averaged over multiple transects and collected by DiFiore et al. (2019). The individual grazing rate ($\alpha$) and the dispersion parameter ($\sigma$), were determined by fitting the Gaussian distribution to this observed data. This process ensures that the herbivores in the model exhibit grazing behavior consistent with the field data, as evidenced by the close alignment between the green dashed line and the solid line in Fig. 1a. Our results show that, when herbivory is accurately represented, the model successfully reproduces the expected spatial patterns of seagrass cover along a transect perpendicular to the reef, as demonstrated by the strong agreement between the model output (purple solid line) and the observed data points (purple dashed line) in Fig. 1a. We observe that seagrass density is lower near the reef, gradually increasing until it reaches a stable density characteristic of a homogeneous meadow. This gradient reflects the herbivores’ preference to remain closer, rather than farther from their reef refuges. When extended to two dimensions, our simulations align closely with satellite imagery of reef halos in the region (Fig. 1b, c), further validating the model$'$s ability to replicate observed landscape patterns.

Notably, the modeled herbivory patterns produce a Gaussian distribution that closely corresponds with the field data (Fig. 1a, green lines), validating the approximations used to derive Eq. (5). As detailed in Supplementary Materials S1, Eq. (5) assumes that $R \gg \sigma$ and $r_c \ll \sqrt{2} \sigma$. For our parameters ($\sigma = 3.98$ m, $R = 40$ m, and $r_c = 1$ m), both conditions hold but with a limited margin. Specifically, $R$ is an order of magnitude larger than $\sigma$, and $r_c$ remains smaller than $\sqrt{2} \sigma$, though not negligible. This suggests that while the approximation is valid, its applicability should be carefully considered. However, the close agreement between simulated and observed grazing patterns indicates that any deviations introduced by these approximations are minimal within this parameter range, supporting the robustness of Eq. (5). Nevertheless, its validity in other parameter regimes should be further examined.

We based our results on a herbivore density of $H = 0.032 \, \text {m}^{-2}$, extracted from surveys conducted by DiFiore et al. (2019) in the study area. For our reef, with an estimated area of $4861 \, \text {m}^2$ and perimeter of $259 \, \text {m}$, this corresponds to an effective perimeter density of $\lambda _H = 0.60 \, \text {m}^{-1}$. While this value is important, the model can still be used without herbivore density data, relying instead on grazing intensity alone. Specifically, the product $A = \alpha \cdot \lambda _H$ can be determined by fitting the Gaussian function in Eq. (5) to measured grazing intensity, along with the dispersion parameter $\sigma$. Here, $A$ represents the total fish grazing intensity per transect, while $\alpha$ corresponds to the individual grazing rate per fish. This observation indicates that halo formation in the model is primarily determined by the total grazing intensity $A$, rather than the specific values of $\alpha$ and $\lambda _H$. For example, using $\alpha = 0.76 \, \text {shoots grazed}\cdot \text {day}^{-1}\cdot \text {fish} ^{-1}$ and $\lambda _H = 6.0 \, \text {m}^{-1}$ yields nearly identical results to those shown in Fig. 1, as the product $A$ remains unchanged. Intuitively, this indicates that the model can replicate observed patterns regardless of whether the exact herbivore density is known, and as long as the overall grazing rate is preserved. However, it is important to mention that in cases where herbivore interactions with seagrass meadows (e.g., through the interaction parameter $\gamma$) or herbivore population dynamics become central to the analysis, careful specification of the fish density $H$ becomes essential to ensure accurate outcomes and conclusions.

### Solution stability

**Fig. 2:**
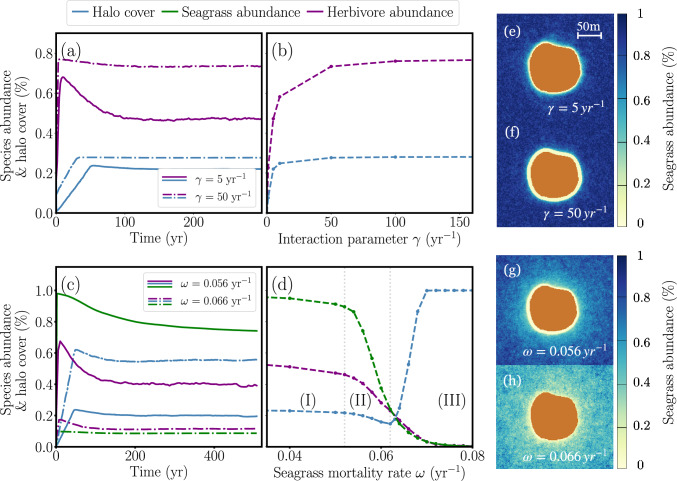
Study of population dynamics and halo cover for different values of the interaction parameter $\gamma$ and seagrass mortality rate $\omega$. Species abundances (green and purple lines) have been calculated from seagrass and herbivore densities, normalized by their maximum values of $2000 \text { m}^{-2}$ and $4 \text { m}^{-2}$, respectively. Halo coverage is represented as the percentage of bare sand in the total available area (blue line). **a**, **b** Analysis of the system’s stability and response to variations in $\gamma$. **c**, **d** Stability and system response to changes in seagrass mortality ($\omega$), with the herbivore–seagrass interaction parameter fixed at $\gamma = 5 \, \text {yr}^{-1}$. The three regions in panel (**d**), separated by the vertical gray dotted lines, represent distinct dynamics of the system: (I) stable halo size despite gradual declines in fish and seagrass populations; (II) slight halo contraction as seagrass decays faster than fish; and (III) halo expansion followed by ecosystem collapse due to severe seagrass loss. **e**–**h** Snapshots of reef halos corresponding to the cases studied in panels (**a**, **c**). The coral reef boundary conditions have been set to a single island of approximately 80 m of diameter. The seagrass growth parameters are set to those of *T. testudinum*, as detailed in Table S1, and the herbivore parameters are fixed as in Table S2, with initial herbivore density set to $\lambda _{H}= 0.60\;\text {m}^{-1}$, and herbivore mortality $\mu = 0.1\;\text {yr}^{-1}$

Simulations reveal that stable halo solutions persist even when herbivore populations are influenced by seagrass availability, interaction governed by the parameter $\gamma$. Building on the Belize case parameters (similar to those in Fig. 1) and introducing a variable herbivore population ($\gamma \ne 0$), we explore the effects of $\gamma$ on system dynamics. The model exhibits stable solutions across different $\gamma$ values, with both halo size and herbivore populations remaining constant over time. This is illustrated in Fig. 2a for $\gamma = 5 \text {yr} ^{-1}$ and $\gamma = 50 \text {yr} ^{-1}$, corresponding to the snapshots in Fig. 2e, f. Figure 2b further illustrates how varying the value of $\gamma$ impacts herbivore abundance and halo area. For low $\gamma$, the fish population fails to grow due to a limited seagrass consumption, leading to extinction and the disappearance of halos. As $\gamma$ increases, herbivores reproduce faster, expanding halo coverage due to greater seagrass consumption. Beyond $\gamma = 50 \text {yr} ^{-1}$, the growth saturates, moderated by the carrying capacity constraint term $(1 - H/H_{\text {max}})$ introduced in “Numerical model” section.

In Fig 2d, we fix $\gamma = 5 \text { yr}^{-1}$ and investigate how changes in seagrass mortality affect the system. We identify three regimes: (I) For $\omega = 0.04 - 0.052 \text {yr}^{-1}$, both fish and seagrass populations gradually decline with increasing seagrass mortality, but halo size remains constant. In this range, the decrease in seagrass density, which would typically lead to larger halos, is counterbalanced by a reduction in fish population, keeping the halo size stable. (II) Beyond this point, seagrass population decays faster than the fish population, causing a slight reduction in halo size in the interval $\omega = 0.052 - 0.062 \text { yr}^{-1}$. (III) When $\omega$ exceeds $0.062 \text { yr}^{-1}$, seagrass density drops so significantly that halos expand until the entire area is bare sand. At this point, the lack of seagrass resources leads to herbivore extinction, resulting in the disappearance of halos. Fig. 2c highlights the stability of population dynamics for representative values of seagrass mortality within regimes (II) and (III), illustrated also by the snapshots in Fig. 2g, h.

### Temperature

**Fig. 3:**
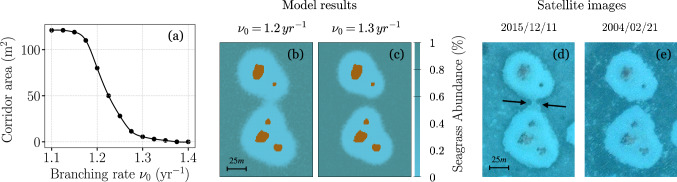
Comparison of model results and field observations for halo size under temperature variations. **a** Empirical halo width from Kāne’ohe, O’ahu, Hawai’i (Innes-Gold et al. 2025) plotted against the average surface water temperature from the 14 days prior to each measurement (black dots), alongside the predicted results from our model (blue solid line). The black dashed line corresponds to a quadratic function that fits the field data: $f(T) = 8.8T -193.3$, with $R^2 = 0.2$. **b** Year-round simulations of halo sizes based on a surface temperature variation as recorded hourly in 2023 by the 51207 NOAA buoy at Moku o Lo’e with hourly precision (red solid line in (**c**)). In this figure, the seagrass mortality and grazing intensity are modeled to depend linearly on temperature (*T*) following the expression: $\omega (T) = 0.45(T- T_0) + 8$, with $T_0 = 24\;^\circ \hbox {C}$. The seagrass growth parameters are set to those of *H. hawaiiana*, as detailed in Table S1, and the herbivore parameters are fixed as specified in Table S2

The model accurately captures the impact of temperature fluctuations on reef halos, successfully reproducing field data from Kāne’ohe Bay, Hawai’i, collected by (Innes- Gold et al. 2025) (Fig. 3). In these simulations, seagrass mortality was assumed to increase linearly with temperature, while fish biomass and grazing intensity remained constant, as outlined in “Field data and model parameters” section. To generate the solid blue line in Fig. 3a, we ran multiple simulations, each at a fixed temperature. The model was allowed to evolve until the halo size reached a steady state, at which point results were compared to observed field data. Specifically, observed halo sizes were plotted against the average temperature measured over the 14 days preceding each field observation (black points in Fig. 3a). Our results (solid blue line) align closely with the curve that fits the field data (dashed black line), capturing the general trend of halo expansion with rising temperature. The simulated increase in halo size with rising temperature is reproduced through a decline in seagrass density, which induces the expansion of the bare sand region.

Given the model’s success in replicating field data, we can also use it to predict seasonal fluctuations in halo size throughout the year, as demonstrated in Fig. 3b. Unlike the simulations in Fig. 3a, where each temperature value was simulated separately and allowed to stabilize, the results in Fig. 3b incorporate hourly variations in seawater temperature, as recorded in Kāne’ohe Bay in 2023 (Fig. 3c). This setup allows the model to simulate dynamic changes in halo size over time, reflecting the seasonal fluctuations in temperature. The comparison between the simulated halo sizes and the observed data provides a robust framework for understanding how real-time environmental changes influence reef halos over the course of the year.

### Corridors connecting reef halos

**Fig. 4:**
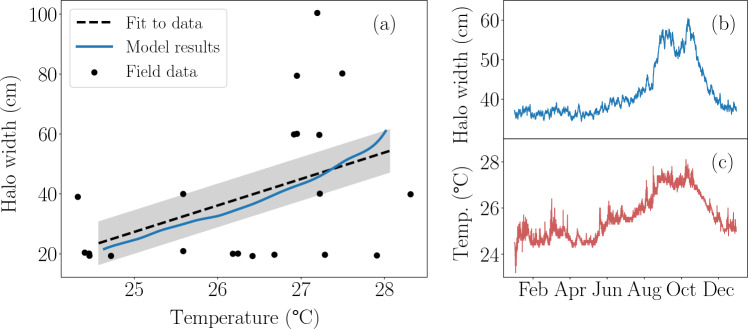
Comparison of model results and field observations of reef halos in Ras Mohammed Natural Park, Egypt. **a** The variation in corridor area as a function of the branching rate ($\nu _0$), averaged over 15 simulations. The corridor area is measured by drawing a 120 m$^2$ rectangle between the two reefs and calculating the area of bare sand within this region. **b**, **c** Model snapshots for branching rates $\nu _0 = 1.2\;\text {yr}^{-1}$ and $\nu _0 = 1.3\;\text {yr}^{-1}$. **d**, **e** Satellite images at coordinates $27^\circ \;44^{\prime }\;31.4^{\prime \prime }\;N, \; 34^\circ \;11^{\prime }\;12.2^{\prime \prime }\;E$ for two different dates, illustrating distinct types of corridors. The seagrass growth parameters are set to those of *H. uninervis*, as detailed in Table S1, and the herbivore parameters are fixed as specified in Table S2

Corridors are linear, vegetation-free pathways that connect adjacent reef halos, interrupting their usual radial symmetry. These distinct spatial structures have been identified in satellite imagery from Ras Mohammed National Park and previously documented by Madin et al. (2022). Our model successfully reproduce these corridor patterns, as demonstrated in Fig. 4. These simulations incorporate dominant seagrass species in the area is *H. uninervis* (Yasser 2009), modeled using its empirically measured parameters and varying slightly around the average reported value for the branching rate $\nu _0$. Corridors clearly emerge at a branching rate of $\nu _0 = 1.2\text{ yr}^{-1}$ (Fig.4b), whereas higher branching rates, such as $\nu _0 = 1.3\text { yr}^{-1}$, inhibit their formation (Fig. 4c). These simulations closely match satellite observations from Ras Mohammed taken on different dates, as shown in Fig. 4d, e. Therefore, our model suggests that the appearance of corridors between reef halos is strongly dependent on the relationship between seagrass branching rate and the distance between halo fronts. This relationship better illustrated in Fig. 4a, where the area of the resulting corridor is plotted as a function of the branching rate. We observe that corridors emerge only when the branching rate $\nu _{0}$ falls below $1.3\text { yr}^{-1}$, with corridor area expanding as $\nu _{0}$ decreases and saturating at around $1.15 \text { yr}^{-1}$.

## Discussion

In this study, we developed the first spatially explicit numerical framework to examine the ecological interactions shaping reef halo formation. By integrating the seagrass growth models proposed by Sintes et al. (2005) with herbivorous fish behavior, our model successfully replicates key patterns such as halo size variability driven by temperature fluctuations (Sec 3.3) and the emergence of grazing corridors (Sec 3.4). In the process, it sheds light on the key mechanisms underlying these phenomena. Despite the complexity of the interactions at play, the model’s simplicity in terms of parameterization makes it an accessible tool for real-world applications. These findings highlight the potential of our framework to deepen our understanding of halo dynamics, provide a foundation for future ecological research, and increase our confidence in the utility of halos as an indicator of coral ecosystem health.

### A novel numerical framework for reef halos

A notable strength of our model is its ability to characterize reef halos using only two data sources: herbivory observations and seagrass growth parameters. This simplicity proves sufficient to replicate observed reef halos in Belize (Fig. 1), capturing essential ecological dynamics. A key aspect of this characterization is the translation of herbivore movement, initially two-dimensional, into one-dimensional grazing data collected along transects (detailed in “Field data and model parameters” section). By fitting a one-dimensional Gaussian distribution to grazing data, our model synthesizes herbivore interactions into measurable parameters, offering a simplified yet robust approach to understanding complex ecological behaviors. The standard deviation ($\sigma$) of this distribution quantifies herbivore movement away from reefs, embedding predator influence via the risk-reward principle without requiring explicit representation of predator dynamics, greatly simplifying the model. Additionally, the amplitude of the Gaussian distribution ($A = \alpha \cdot \lambda _H$) is related to the total grazing intensity per transect. To complete the parameterization, seagrass growth is parameterized using average values compiled in Marbà and Duarte (1998), facilitating application across different species.

Our spatially explicit approach represents a significant advancement over previous methods, such as the non-spatial consumer-resource model by Ong et al. (2025). Using partial differential equations with geometric considerations, their work effectively examines how reef configurations shape halo sizes while avoiding the complexity of explicitly modeling spatial processes. However, halos are inherently spatial phenomena, driven by localized herbivore behavior that disrupts seagrass growth near coral reefs. These spatial dynamics can generate distinctive patterns, such as grazing corridors, which break the typical radial symmetry of halos, necessitating detailed spatial modeling to fully capture their emergence. Our framework addresses this by modeling herbivore movement through an agent-based approach, which is then coupled to seagrass dynamics. Despite the methodological differences, both models share conceptual similarities in their focus on interactions among herbivores and vegetation. See Supplementary Materials S4 for a detailed comparison, including how the homogeneous partial differential equation version of our model aligns with the equations presented by Ong et al. (2025).

### Persistence and variability in reef halos

Halos represent stable ecological structures that reflect the balance and dynamic interplay between predators, herbivores, and primary producers such as seagrass (DiFiore et al. 2019; Madin et al. 2022). Herbivores graze on seagrass, yet their movement is largely confined to reef-adjacent areas due to the risk of predation that increases with distance from reef shelter. The total seagrass consumed is influenced by herbivore population dynamics, both this behavioral response to risk and herbivore population dynamics, which in turn depend on the availability of seagrass. This intricate feedback loop governs both the size and persistence of halos. Despite the complex interactions involved, halos are known to endure over long timescales of at least half a century (Madin et al. 2022). Our model captures this stability by incorporating the parameter $\gamma$, which quantifies the strength of the herbivore–seagrass interaction, as shown in Fig. 2. Although $\gamma$ is not directly measurable, unlike herbivore dispersion $\sigma$ or grazing rate $\alpha$, it provides valuable insights into the dynamics of fish–seagrass interactions.

Even though reef halos persist over extended periods, their sizes have been reported to fluctuate (Madin et al. 2022). Such oscillations were predicted in previous models by Ong et al. (2025) under fixed environmental conditions, such as constant temperature and nutrient levels. Their solutions suggest that dispersed coral reef distributions allow herbivores more uniform shelter from predators, enabling overgrazing and periodical fluctuations in halo patterns. Although our model does not currently predict such periodicities under fixed environmental conditions, incorporating a term such as $SH/(1+S)$ to our interactions could replicate these dynamics. This functional form, rooted in the Rosenzweig–MacArthur model (Rosenzweig and Macarthur 1963), is central to the oscillatory behavior in Ong et al. (2025)’s framework (see Supplementary Materials S4). Because the oscillations arise from this nonlinear interaction term, even small parameter perturbations can push the system from steady state into a cyclic behavior. By extending our model with such terms, we could explore oscillatory halo dynamics in a spatially explicit context.

In our framework, halo size variability is driven mainly by external factors—most notably seasonal temperature changes. While periodic halos could theoretically arise under constant conditions after a perturbation, field studies show that real halos fluctuate with environmental variability instead (Innes-Gold et al. 2025; Lester et al. 2025). Unlike the intrinsic oscillations predicted by Ong et al. (2025), these fluctuations cannot be explained by herbivory and risk alone; other biotic and abiotic factors clearly contribute (Madin et al. 2019a; Bilodeau et al. 2021). Our model provides an ideal framework for integrating these diverse influences, offering a comprehensive approach to understanding halo size variability. For example, observations in Kāne’ohe Bay (Innes-Gold et al. 2025) show that halos expand with rising temperatures, likely due to temperature-driven declines in vegetation growth. Our simulations in Fig. 3 support the hypothesis that the increase in halo size with rising temperature can be explained primarily by the decline in seagrass density, which leads to the expansion of the bare sand region, or halo.

Although our model reproduces the observed halo variability in Kāne’ohe Bay, additional data is needed to fully understand the underlying dynamics. Existing field data shows no temperature correlation with either herbivore biomass or nutrient availability, but they do not provide information on how grazing intensity varies with temperature (Innes-Gold et al. 2025). Because higher temperatures accelerate fish metabolism and can elevate per-capita herbivory rates (Nagelkerken et al. 2023; Volkoff and Ronnestad 2020), this omission could mask an important driver of halo expansion. In Fig. 3, grazing intensity is treated as temperature-independent, so all halo size variation is attributed to the temperature sensitivity of shoot mortality. In the Supplementary Materials S3, we explore an alternative parametrization that assumes that shoot mortality and grazing intensity rise with temperature, providing an equally plausible explanation for the observed pattern. The two parameterizations match the field data equally well (see Fig. 3 and Fig. S4), which highlights the need for targeted measurements—such as temperature-dependent bite-rate assays and grazer-exclusion experiments that quantify shoot mortality—to disentangle herbivory from vegetation decline and resolve the mechanisms underlying halo variability.

While vegetation cover plays a central role in shaping halo dynamics, its influence varies across systems depending on the species involved and their response to environmental conditions (Bilodeau et al. 2021). For example, in Ningaloo, Australia, macroalgae-dominated habitats exhibit decreased halo sizes during warmer months due to increased macroalgal productivity (Lester et al. 2025). This contrasts with the dynamics observed in Kāne’ohe Bay (Innes-Gold et al. 2025), where halos expand with rising temperatures, driven by seagrass decline. Such divergent behaviors highlight the importance of comparative studies to uncover the broader implications of environmental fluctuations on halo formation and persistence. Accordingly, extending temperature-dependent simulations to other study sites—such as Belize or Egypt—requires site-specific data, because differences in species composition, thermal regimes, and trophic interactions mean that parameters calibrated for Hawai’i may not hold. Our model nonetheless offers an ideal framework for these investigations, as it can be parametrized to account for different seagrass species, grazing behaviors, and environmental factors, enabling targeted studies across ecosystems.

### Uncovering the mechanisms behind reef halo corridors

Our model provides new insights into previously unexplained behaviors in halos, particularly the formation of reef halo corridors—linear, vegetation-free paths that connect two otherwise separated halos and disrupt their usual radial symmetry. Our model successfully reproduces these corridors (see “Corridors connecting reef halos” section), which is a significant validation of its ability to simulate complex spatial dynamics. Notably, the corridors emerge spontaneously within the model, without the need for explicit implementation, highlighting its capacity to capture realistic ecological processes.

Corridor-like structures between halos were first documented by Madin et al. (2022), but the mechanisms behind their formation remained unresolved. While Madin suggested they might result from shifts in herbivore foraging behavior, we propose instead that these patterns are better explained by the rhizomatic clonal growth of seagrasses. Seagrasses grow radially, with new shoots emerging uniformly from apices in all directions, especially in the central regions of the meadows. However, between two halos nearing convergence, shoot recruitment occurs predominantly along specific directions. This directional bias arises because heavily grazed areas within halos lack viable apices for new shoot formation; consequently, new growth is channeled from areas experiencing lower grazing pressure. The dominant direction of shoot recruitment is illustrated by the black arrows in Fig. 4. These arrows, oriented roughly perpendicular to the corridor’s path, indicate the most probable trajectories along which shoots can successfully grow without being grazed.

As halos expand and their boundaries converge, the probability of shoot recruitment at points between them decreases, leading to the spontaneous emergence of corridors. This phenomenon is influenced by the seagrass branching rate, which controls how frequently rhizomes split before reaching the grazing front. Lower branching rates result in fewer ramification events—i.e., instances where rhizomes split to form new offshoots—increasing the likelihood of bare sand corridors persisting between halos. Model results (Fig. 4) support this hypothesis, showing that higher branching rates suppress corridor formation. Importantly, corridor emergence is not unique to the Ras Mohammed configuration; our model predicts similar structures in other landscapes. For instance, in the Belize scenario depicted in Fig. 1, increasing the value of the branching rate leads to the development of corridors connecting previously separated halos (see results in Fig. S5), which reinforces the generality of the proposed mechanism. These findings highlight the value of numerical models in uncovering the ecological processes that shape spatial patterns in marine ecosystems.

Observations from Ras Mohammed reveal fluctuations in the prevalence, size, and shape of corridors over time (Madin et al. 2022). Our results suggest that such variability may be linked to temporal changes in environmental conditions that influence seagrass branching rates. While our study assumes constant branching rates, small variations—such as those between 1.2 and $1.3\,\text {yr}^{-1}$—could plausibly arise from factors like nutrient availability, hydrodynamic changes, or localized disturbances (Duarte and Sand-Jensen 1990; Vidondo et al. 1997). These subtle shifts may have significant implications for corridor dynamics (Fig. 4), emphasizing the need for further investigation into how environmental variability affects seagrass growth and branching rates in these regions.

Our simulations suggest that corridor formation is tightly linked to the rhizomatic growth strategy typical of seagrasses. Based on this, we infer that macroalgal beds relying on spore dispersal for growth, rather than directional rhizome extension, are unlikely to exhibit similar corridor structures. However, little is known about whether macroalgae-dominated habitats support corridor formation, as the literature on this topic is sparse to non-existent. While identifying corridors in satellite images is relatively straightforward, determining the species composition of these habitats is more challenging, particularly since in situ site exploration is often required to do so. To address this gap, a broader survey of corridor development across different ecosystems is needed, integrating global satellite imagery with species identification and branching rate measurements. This approach would provide robust evidence to either support or refute the mechanisms for corridor formation proposed in this work.

### Expanding the scope of reef halo models

Although macroalgal meadows are generally less widespread and extensive than seagrass meadows, they are still ecologically significant (Waycott et al. 2009; Duarte et al. 2018). Some macroalgae, such as *Caulerpa*, exhibit rhizomatic growth, which aligns well with our current modeling framework (Llabrés et al. 2022). Other species, like *Sargassum*, form meadows through spore-based reproduction and can also exhibit reef halos (Lester et al. 2025), hinting at the broader occurrence of this phenomenon across different ecosystems. For instance, halos are not limited to aquatic environments: in terrestrial forests, beavers create tree-removal halos around ponds due to predation risk posed by wolves (Gable et al. 2023), and herbivores like rabbits, rodents, and birds may generate halos around grassland shrubs (Bartholomew 1970). To better capture such dynamics, developing a more generalized model of herbivore–vegetation interactions that incorporates both rhizomatic and non-rhizomatic growth strategies would expand the scope and applicability of our simulations. Moreover, while this study focuses on mono-specific seagrass meadows, the modeling framework is readily adaptable to mixed meadows with interspecific interactions, as demonstrated in prior works (Llabrés et al. 2022, 2023; Moreno-Spiegelberg and Gomila 2024).

A critical next step in broadening our framework is to recognize that halo size responds not only to natural grazer–vegetation feedbacks but also to anthropogenic pressures such as eutrophication and fishing. Nutrient enrichment increases epiphytic loading and reduces light penetration, ultimately depressing seagrass growth and altering the balance between rhizomatic and non-rhizomatic primary producers (Burkholder et al. 2007; Lapointe et al. 2019). This effect can be captured by introducing a nutrient-dependent forcing term that depresses intrinsic seagrass branching rates $\nu _0$ when nutrient thresholds are surpassed, thereby reproducing halo expansion under progressive eutrophication. Also, fishing efforts influence halo geometry both directly, by removing herbivorous fish and invertebrates, and indirectly, by altering predator abundance and hence grazer risk sensitivity (Madin et al. 2019a). These effects can be reflected by introducing a harvest term in the herbivore density and by allowing the grazer behavior parameter $\sigma$ to vary as a function of predator density, which itself responds to fishing pressure. By explicitly incorporating these human-driven stressors, the model can convert observed halo patterns into quantitative diagnostics of ecosystem condition and provide a virtual test to evaluate mitigation options.

With future refinements, the potential of our model extends beyond the ecological study of the complexities behind reef halos. Our goal is to make this model accessible and adaptable to a broad range of users. Reef halos offer a scalable, cost-effective solution for early diagnostics of ecosystem health and habitat degradation (Madin et al. 2022). By integrating AI-driven tools for halo identification (Franceschini et al. 2023) within our framework’s interface, we could significantly enhance the utility of halos as valuable tools in ecological research and conservation. Our goal is to make this model accessible and adaptable to a broad range of users. To achieve this, we aim to develop an intuitive, interactive tool—a resource that conservationists, researchers, and even local communities can use to simulate reef halo dynamics across diverse ecosystems. This tool would enable users to adjust key parameters, such as herbivore density, seagrass growth rates, and grazing intensities, to model grazing patterns tailored to their specific study areas.

## Supplementary Information

Below is the link to the electronic supplementary material.

Supplementary file 1 (pdf 2764 KB)

## Data Availability

The code for simulation in this article is published in Zenodo with DOI 10.5281/zenodo.16208588. The field data used in this article is already published in other peer-reviewed articles (DiFiore et al. 2019; Innes-Gold et al. 2025).

## Code availability

The model software used for the simulations presented in this article is openly available on Zenodo (Llabres et al. 2025, DOI 10.5281/zenodo.16208588). The field data analyzed here were previously published in peer-reviewed studies (DiFiore et al. 2019; Innes-Gold et al. 2025).
