# Supplementary material for: A spatial numerical model for seagrass–herbivore interactions and the formation of reef halos
Source: Coral Reefs. 2025 Aug 20;44(5):1587–99. doi: 10.1007/s00338-025-02729-3 (PMC12500768; doi:10.1007/s00338-025-02729-3)
Supplement: Supplementary file 1 — (pdf 2764 KB) [file 338_2025_2729_MOESM1_ESM.pdf]

Supplementary Material: A spatial numerical model for seagrass-herbivore interactions and the formation of reef halos

Eva Llabrés[1,2], Anne A. Innes-Gold[2], Bartholomew DiFiore[3], Tomàs Sintes[1], Elizabeth Madin[2]

[1]Institute for Cross-Disciplinary Physics and Complex Systems (IFISC), CSIC-UIB, Palma de Mallorca, 07122, Spain.
[2]Hawaii Institute of Marine Biology, University of Hawai'i at Mānoa, Kāne'ohe, HI, 96744, USA.
[3]Gulf of Maine Research Institute, Portland, Maine, 040101, USA.

S1 Herbivore dynamics: 2d to 1d simplification

In Sec. 2.1 of the main text, we explore the dynamics of seagrass mortality attributed to grazing, which is quantified through the implementation of the following expression:

$$\mathcal{F}(\vec{x}) = \frac{\alpha}{2\pi\sigma^2} \sum_{n \in N_H} e^{-\frac{(\vec{x}-\vec{x}_n)^2}{2\sigma^2}} , \tag{S1}$$

where the summation of n encompasses the total number of herbivores N_H, with each $\vec{x}_n$ representing the 2-dimensional coordinates of their respective refuges at the perimeter of the reef. In this section, we will reduce the formula of equation (S1) to an expression in 1d, since we usually measure along transects perpendicular to the reef perimeter. For simplicity, we will consider the transect that goes along horizontal axis, which can be characterized by $\vec{x} = (x, 0)$, where x represents the distance measured from the reef perimeter. See Fig. S1 for a graphical representation. For this calculation, we will assume a fairly circular patch reef - common feature among many small reefs (Giménez-Romero et al. 2024) - with radius R. If the reef is sufficiently large, we can consider σ to be small compared to the radius of the reef ($R \gg \sigma$), allowing us to approximate the perimeter as a straight line. The value σ serves as a threshold, as the refuges located farther than σ from the origin of the transect will contribute insignificantly to the sum in equation (S1) due to the rapid decay of the Gaussian

function. Therefore, the refuges will coincicide with the vertical axis, as also illustrated in Fig. S1, and they will have only have y-components in their coordinates, i.e. $\vec{x}_n = (0, y_n)$. With this considerations in mind, we can rewrite equation (S1) as:

$$\mathcal{F}(x) \sim \frac{\alpha}{2\pi\sigma^2} e^{-\frac{x^2}{2\sigma^2}} \sum_{n \in N_H} e^{-\frac{{y_n}^2}{2\sigma^2}} \,. \tag{S2}$$

We now have a one-dimensional formula. To simplify and make it more compact, we evaluate the sum. For this purpose, we consider that the model operates on a lattice with a side length of r_c. Assuming a constant linear density of fish along the perimeter, denoted as λ_H [m^{-1}], the sum over individual fish can be reduced to a sum over the perimeter lattice cells, where $y_n = n \cdot r_c$ with $n \in \mathbb{Z}$:

$$\mathcal{F}(x) \sim \frac{\alpha \cdot \lambda_H \cdot r_c}{2\pi\sigma^2} e^{-\frac{x^2}{2\sigma^2}} \sum_{n \in \mathbb{N}} e^{-\frac{(n \cdot r_c)^2}{2\sigma^2}} \tag{S3}$$

If the lattice cells are sufficiently small, i.e., $r_c << \sqrt{2}\sigma$, we can approximate the discrete sum by a continuous integral. Introducing $z = n \cdot r_c$, we transition to the continuum:

$$\mathcal{F}(x) \sim \frac{\alpha \cdot \lambda_H}{2\pi\sigma^2} e^{-\frac{x^2}{2\sigma^2}} \int_{-\infty}^{\infty} e^{-\frac{z^2}{2\sigma^2}} \, dz \quad = \quad \frac{\alpha \cdot \lambda_H}{\sqrt{2\pi}\sigma} e^{-\frac{x^2}{2\sigma^2}} \,, \tag{S4}$$

where in the last equality we have used the result of the integral $\int_{-\infty}^{\infty} dz \, e^{-z^2} = \sqrt{\pi}$ for the Gaussian normalization. It is useful to define the quantity $A = \alpha \cdot \lambda_H$ [shoots grazed $\cdot$ yr^{-1} $\cdot$ m^{-1}], as it represents the total grazing rate per unit length. This term quantifies the combined grazing pressure on the seagrass meadow along a transect perpendicular to the reef perimeter, encapsulating the combined effect of herbivore density and grazing intensity along each transect.

This expression is key to characterizing fish grazing and isolating the average behavior of individual fish. By assuming a roughly circular patch reef, symmetry suggests that all transects yield similar results. However, it is important to note that patch reefs are rarely perfectly circular, and herbivore density may vary significantly. To address these variations, our analysis uses data averaged across multiple transects, making the approximation sufficiently robust for practical purposes. While the model incorporates some irregularities, these assumptions primarily serve to establish a baseline for average grazing behavior among fish that aligns with observed data.

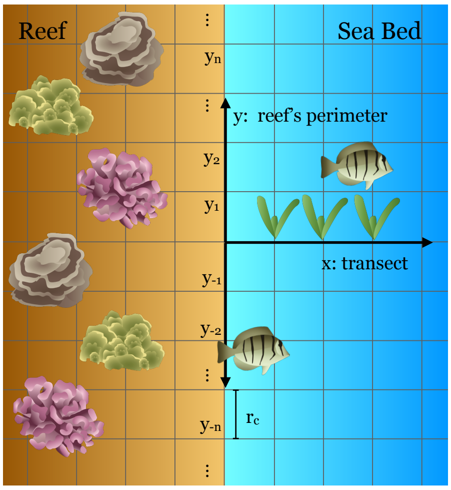

Fig. S1: Schematic representation of the mathematical derivation to reduce the grazing mortality formula from two to one dimension. The diagram illustrates a transect along the horizontal axis (x), perpendicular to the reef's perimeter (y). Herbivore refuges are positioned along the vertical axis ($y_n = n \cdot r_c$), representing lattice cells with spacing r_c. Schematic representation created using the vector graphics editor *Vecta.io*.

S2 Seagrass mortality from field data

Field data collected by Innes-Gold et al. (in press) in Kāne'ohe Bay are presented in Fig. S2. Panel S2a displays scattered data, which reveals a weak correlation between herbivore biomass and temperature, as reflected by a coefficient $R^2 = 0.001$. In our model all grazing fishes are treated as ecologically equivalent: every individual removes vegetation at the same rate, regardless of species or size. Herbivore density therefore represents an average grazer count per square meter. In this case, the total grazing pressure scales directly with biomass, and because the field data indicate that biomass does not vary with temperature (Fig. S2a), we set $\gamma = 0$, keeping the number of individuals constant across simulations with different temperatures.

In Fig. S2b, a linear decline in seagrass cover with increasing temperature is observed, with a slope of $-0.041\,^\circ\mathrm{C}^{-1}$ and $R^2 = 0.15$. This indicates that for every 1°C rise in temperature, seagrass cover decreases by 0.041 units. The model further reveals a linear relationship between seagrass cover and seagrass mortality, shown in Fig. S3, with a slope of $-0.092\,\mathrm{yr}$. This means that for every unit increase in the mortality rate, seagrass cover decreases by 0.092 units. By combining these two observations, we can deduce how temperature affects shoot mortality:

$$a = \frac{\text{slope of seagrass cover vs. temperature}}{\text{slope of seagrass cover vs. mortality}} = \frac{-0.041}{-0.092} = 0.45\,\mathrm{yr}^{-1} \cdot {}^\circ\mathrm{C}^{-1}.$$

This result suggests that seagrass shoot mortality increases linearly with temperature at a rate of $a = 0.45\,\text{yr}^{-1}$ for every 1°C rise.

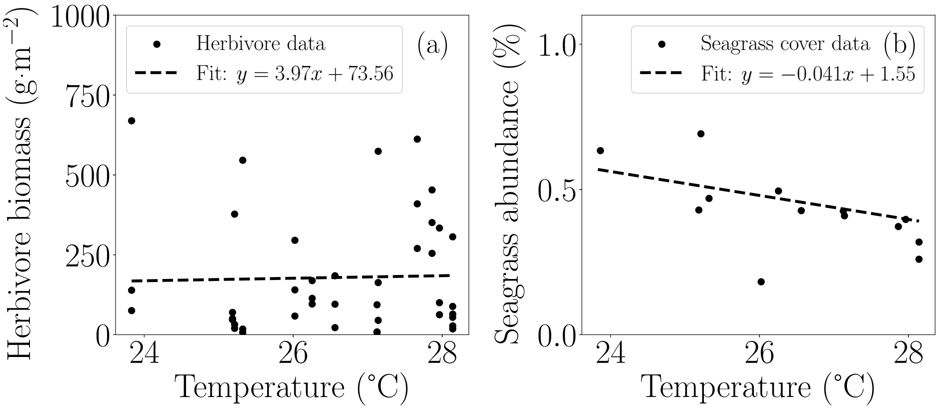

Fig. S2: Field data collected in the Kāne'ohe Bay by Innes-Gold et al. (in press) (a) Relationship between herbivore biomass and temperature. The data were fitted to a linear equation, yielding a correlation coefficient of $R^2 = 0.001$. Measurements were taken at sites distant from (artificial) reefs, where herbivory is assumed to be minimal. (b) Seagrass cover against the average surface water temperature from the 14 days prior to each measurement. The linear fit has a correlation coefficient of $R^2 = 0.15$.

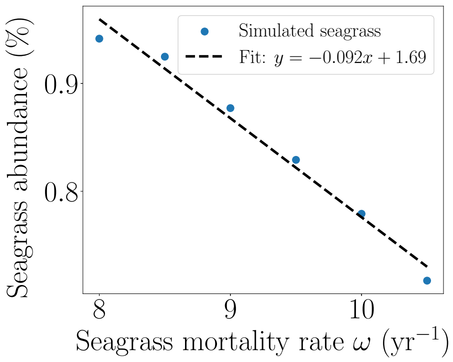

Fig. S3: Model results (blue dots) for the dependence of seagrass abundance of *Halophila hawaiiana* on variations in the shoot mortality parameter, ω. The linear fit (black dashed line) has a correlation of $R^2 = 0.98$.

S3 Alternative simulations for temperature dependent halos

Figure 3 in the main text analyses the effect of temperaturedriven shoot mortality on halo size by allowing only the mortality rate

$$\omega(T) = 0.45\,(T - T_0) + 8, \qquad T_0 = 24°\mathrm{C}, \tag{S5}$$

to vary linearly with sea-surface temperature T, while the grazing intensity $\alpha =$ is fixed. This choice follows the rationale in Sec. S2: mortality coefficients were inferred from vegetation-change experiments that did not exclude herbivores, making it impossible to isolate any temperature effect on grazing. To test whether rising temperature might also amplify herbivore consumption, Fig. S4 adopts an alternative parameterisation in which both seagrass mortality and grazing intensity respond to temperature

$$\omega(T) = 0.041\,(T - T_0) + 7\,, \qquad \alpha(T) = 0.10\,(T - T_0) + 0.138, \tag{S6}$$

again with $T_0 = 24°$C. All other parameters are identical to those used for Fig. 3: herbivore dispersion $\sigma = 0.42\,\mathrm{m}$, constant herbivore density $\lambda_H = 4.2\,\mathrm{m}^{-1}$, and the growth traits of *H. hawaiiana* listed in Table S1.

Figures 3a and S4a present the halo widths predicted by the model (solid blue lines) and the empirical measurements performed by Innes-Gold et al. (in press) (black dots). In each panel, the dashed black curve represents an independent statistical fit to the field datalinear in Fig. 3a and quadratic in Fig. S4a, chosen to illustrate two equally plausible empirical relationships. Despite their different functional forms, the two regressions achieve nearly identical goodness of fit ($R^2 \approx 0.180.20$), and in both cases the results of the simulations fall entirely within the grey uncertainty envelope of the observations. This concordance implies that the present data cannot, on their own, discriminate between the only temperature-dependent seagrass-mortality model of equation (S5) and the coupled mortality-plus-grazing model of equation (S6). Resolving the dominant mechanism will therefore demand dedicated field campaigns that independently measure temperature-driven shoot mortality and grazing intensity, supplying the empirical constraints required for robust parameterisation of the model.

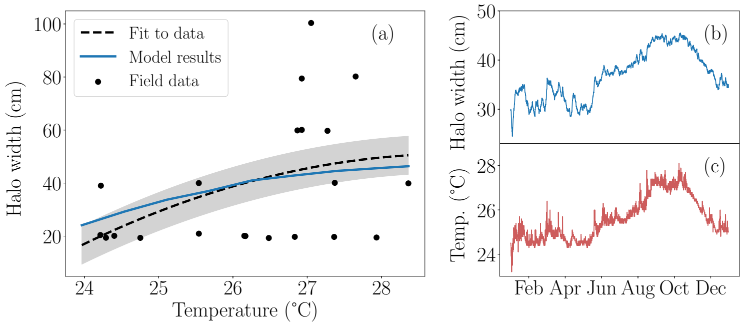

Fig. S4: Comparison of model results and field observations for halo size under temperature variations. In this figure, the seagrass mortality and grazing intensity are modeled to depend linearly on temperature, T, following the expressions: $\omega(T) = 0.041(T - T_0) + 7$ and $\alpha(T) = 0.1(T - T_0) + 0.138$, with $T_0 = 24$°C. (a) Halo width recorded by Innes-Gold et al. (in press), plotted against the average surface water temperature from the 14 days prior to each measurement (black dots), alongside the predicted results from our model (blue solid line). The black dashed line corresponds to a quadratic function that fits the field data: $f(T) = -1.4T^2 + 78.1T - 1085.5$, with $R^2 = 0.18$. (b) Year-round prediction of halo size variation based on (c) surface temperature recorded in 2023 by the 51207 NOAA buoy in Moku o Lo'e with hourly precision (red solid line). The herbivore dispersion is set to $\sigma = 0.42\,m$, and the herbivore number is assumed constant ($\gamma = 0, \lambda_H = 4.2\text{m}^{-1}$). The seagrass growth parameters are fixed as those of *H. hawaiiana*, collected in the Supplementary Materials S5.

S4 Mathematical formulation and comparison of reef halo models

The interactions described in our model (Sec. 2.1) can also be expressed in terms of partial differential equations. Considering only the homogeneous case, where spatial dependence is absent, the relevant interactions between seagrasses (S) and herbivores (H) are given by:

$$\frac{dS}{dt} = \alpha S\,(1 - S) - \alpha \mathcal{F}(S, \sigma) S + (\nu_0 - \omega) S, \tag{S7}$$

$$\frac{dH}{dt} = \gamma \mathcal{G}(S, \sigma) H\,(1 - H) - \mu H, \tag{S8}$$

where we normalize $S = S/S_{\text{max}}$ and $H = H/H_{\text{max}}$. The spatially dependent functionals were originally defined as:

$$\mathcal{F}(H, \sigma, x) = \frac{1}{2\pi\sigma^2} \sum_{i \in N_H} e^{-\frac{(\vec{x} - \vec{x}_i)^2}{2\sigma^2}}, \qquad \mathcal{G}(S, \sigma, x) = \frac{1}{2\pi\sigma^2} \int d\vec{x}' e^{-\frac{(\vec{x}' - \vec{x})^2}{2\sigma^2}} S(x'), \tag{S9}$$

where i iterates over the N_H herbivores, and $\vec{x}_i$ represents their shelter locations. To derive a fully homogeneous version of equations (S7)-(S8), the spatially dependent

functionals $\mathcal{F}$ and $\mathcal{G}$ should be replaced by constants that reflect the average grazing intensity and average seagrass biomass across the system, respectively. This step effectively removes spatial variability from the equations.

For comparison, we include the equations proposed by Ong et al. (2025), which describe reef halos using a mean-field consumer-resource model without explicit spatial dependence:

$$\frac{dS}{dt} = \alpha S \left(1 - \frac{S}{S_0 e^{-R(S_0 - S)}}\right) - \alpha \mathcal{J}(S, g) S, \tag{S10}$$

$$\frac{dH}{dt} = \gamma \mathcal{J}(S, g) H \left(1 - H\right) - \mu H, \tag{S11}$$

where the functional $\mathcal{J}(S, g) = \frac{S}{1+gS}$ is inspired by the Rosenzweig-MacArthur model (Rosenzweig and Macarthur 1963) and introduces periodic population dynamics without the need of environmental seasonal variability. The term $S_0 e^{-R(S_0 - S)}$ implicitly accounts for the geometry of reef distribution. This clever simplification eliminates the need to explicitly model spatial effects while still capturing some geometric constraints.

S5 Supplementary tables and figures

	Spacer length	Rhizome elongation	Branching rate	Branching angle	Mortality	Max. density
Symbol	δ	v	ν_0	ϕ	ω_0	S_{max}
Units	cm	cm yr^{-1}	branches yr^{-1}	degree	shoots yr^{-1}	shoots m^{-2}
T. testadinum	7.0	69.0	0.07	36.3	0.05	2000
H. hawaiiana	1.7	89.0	10.3	59.5	7	4000
H. uninervis	2.7	101.0	1.3	57.2	1.1	1100

Table S1: Clonal growth parameters for the seagrass species *Thalassia testadinum*, *Halophila hawaiiana*, and *Halodule uninervis*. Their values are found by direct experimental observation, we refer to the review by Marbà and Duarte (1998).

	Grazing rate	Herbivore dispersion	Linear fish density	S-H interaction	Fish mortality
Symbol	α	σ	λ_H	γ	μ
Units	shoots grazed $\cdot$ day^{-1} $\cdot$ $fish^{-1}$	m	m^{-1}	yr^{-1}	yr^{-1}
Belize	7.6	3.98	0.6	0-150	0-0.1
Hawai'i	0.14	0.42	4.2	0	0
Egypt	1.62	10.38	25.0	0	0

Table S2: Key herbivore parameters in our model and the values used to set them in our simulations.

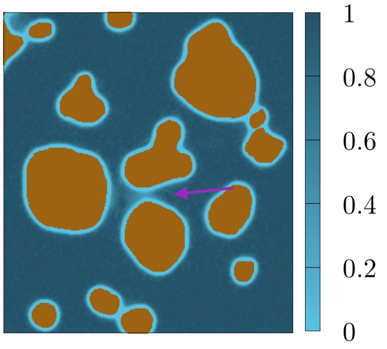

Fig. S5: Simulation of grazing patterns around patch reefs in Belize using our model increasing the branching rate value to ($\nu_0 = 0.07yr^{-1}$) respect the simulations performed Fig. 1c ($\nu_0 = 0.09yr^{-1}$). The purple arrow shows a newly emergent corridor that appears when increasing the branching rate. The color bar represents percentage of seagrass cover with colors ranging from light blue (low density) to dark blue (high density). Patch reefs are depicted in brown. Parameters for seagrass growth are based on *T. testadinum* and detailed in Table S1, except the branching rate which is fixed to ($\nu_0 = 0.09yr^{-1}$). Herbivore parameters are fixed as in Table S2, with $\gamma = 0$ for constant herbivore density.

References

Giménez-Romero À, Matías MA, Duarte CM. Unravelling the Universal Spatial Properties of Coral Reefs. Global Ecology and Biogeography. 2024;p. e13939. https://onlinelibrary.wiley.com/doi/abs/10.1111/geb.13939, e13939 GEB-2024-0139.R4, https://doi.org/https://doi.org/10.1111/geb.13939. https://onlinelibrary.wiley.com/doi/pdf/10.1111/geb.13939.

Innes-Gold AA, Mcmanus L, Lester E, Ong T, Cook-McNab A, Rahnke S, et al. Coral reef halo dynamics are driven by herbivory and temperature. The American Naturalist,. in press;accepted with minor revision.

Marbà N, Duarte C. Rhizome elongation and seagrass clonal growth. Marine Ecology Progress Series. 1998;174:269–280. https://doi.org/10.3354%2Fmeps174269, https://doi.org/10.3354/meps174269.

Ong TW, McManus LC, Vasconcelos VV, Yang L, Su C. Seeing Halos: Spatial and Consumer-Resource Constraints to Landscapes of Fear. The American Naturalist. 2025 Jun;205(6):590–603. http://dx.doi.org/10.1086/735688, https://doi.org/10.1086/735688.

Rosenzweig ML, Macarthur RH. Graphical Representation and Stability Conditions of Predator-Prey Interactions. The American Naturalist. 1963;97:209 – 223. https://api.semanticscholar.org/CorpusID:84883526.
